# Cross-cultural translation and adaptation of Functional Assessment of Cancer Therapy – Multiple Myeloma tool – MM1 and LEU3 – for Portuguese

**DOI:** 10.31744/einstein_journal/2022AO4457

**Published:** 2022-01-27

**Authors:** Juliana Todaro, Polianna Mara Rodrigues de Souza, Marci Pietrocola, Fernanda da Cunha Vieira, Nazaré Solange da Silva Amaro, Jandey da Gloria Bigonha, José Belém de Oliveira, Auro del Giglio

**Affiliations:** 1 Hospital Israelita Albert Einstein São Paulo SP Brazil Hospital Israelita Albert Einstein, São Paulo, SP, Brazil.; 2 Centro Universitário FMABC Santo André SP Brazil Centro Universitário FMABC, Santo André, SP, Brazil.

**Keywords:** Quality of life, Multiple myeloma, Surveys and questionnaires

## Abstract

**Objective:**

To describe of the translation from English to Portuguese and adaption process of subitems of the Functional Assessment of Cancer Therapy *–* Multiple Myeloma.

**Methods:**

In the first phase, translations from English into Portuguese of two subitems of Functional Assessment of Cancer Therapy *–* Multiple Myeloma were performed. Subsequently, a consensus and back translation were conducted, and, finally, translation and back translations were reviewed by four independent bilingual experts. In the second phase, the translated subitems were applied, along with a questionnaire, to 10 native Portuguese speakers patients with multiple myeloma.

**Results:**

There was a recognition of the translation process in its first version applied to 10 patients with multiple myeloma, whose reported no difficult to understand the translated and validated instrument. Patients also did not find the content irrelevant or offensive, and they did not suggested changes.

**Conclusion:**

The subitems of the Functional Assessment of Cancer Therapy *–* Multiple Myeloma were translated from English into Portuguese following the proposed methodology and there was not need of readjustments. This process allowed this instrument of quality of life, which is widely known to be beneficial in the management of patients with multiple myeloma, to be used among our population.

## INTRODUCTION

Multiple myeloma (MM) is defined as a hematological neoplasm characterized by the proliferation of clonal plasma cells secreting immunoglobulin in the blood and urine (M protein). This disease incidence is estimated to account for 1% of all neoplasia. Multiple myeloma affects 4.5 cases/100 inhabitants per year, and accounting for 10% of all hematological neoplasms. In Brazil, there are no accurate statistics on incidence of MM provided by the records of the National Cancer Institute José Alencar Gomes da Silva (INCA - *Instituto Nacional de Câncer José Alencar Gomes da Silva*).^(1-3)^

The MM is a disease of the elderly and the mean age of its diagnosis is 70 years. Of diagnosed cases 1% is under 40 years and 50% over 65 years. In Brazil, the mean age at diagnosis is 10 years lower than those reported in published literature, and this fact can be explained given that the most of diagnoses occur at advanced stages.^(4,5)^

The clinical presentation of MM is variable, and the chain of myeloma development, which possibly begins from monoclonal gammopathy, may be mediated by a condition called “smoldering myeloma”. This, as suggested by its name, does not present symptoms despite the presence of 10% plasma cells and evidence of a monoclonal protein (diagnostic criterion).^(1)^

Classically, MM, in its symptomatic form, has its main manifestations grouped under the acronym CRAB, which stands for hypercalcemia (calcium), renal failure, anemia, and bone lesions.^(1)^In addition to the symptoms already related and secondary to the target organ lesion, many patients with MM complain of fatigue, even in the asymptomatic phase.^(6)^

Cancer-related fatigue is not clearly defined. The National Comprehensive Cancer Network (NCCN) classifies it as a stressful, persistent, subjective feeling of tiredness or emotional, cognitive, and physical exhaustion related to cancer or its treatment. In addition, cancer-related fatigue is recognized as not related to individual’s recent activity, or not capable of interfering with their usual activities.^(7)^

In cancer disease, fatigue is considered multicausal, *i.e.*, it is not only associated with the neoplastic process, but also to the therapy used and the comorbidities (physical and emotional). The importance of fatigue is also highlighted because it is a persistent symptom, which can continue after the end of treatment and may affect negatively the continuity of treatment.^(8)^

Conceptually, MM is treated in the symptomatic form, however, this is debatable for patients in the smoldering phase stratified as high risk, considering the speed of progression to the symptomatic disease.^(9)^

The myeloma treatment model is derived from the importance given, until the 1990s, to autologous bone marrow transplantation for young patients. This was because the use of alkylating agents was affected due to the impairment of the mobilization of hematopoietic cells in the pre-transplant phase, and at that time, melphalan was a major chemotherapeutic agent. For this reason, although sometimes the role of transplantation has been questioned, it is still used as a consolidation, and patients are still divided by their transplant eligibility criteria for the choice of the therapy.^(9)^

Coleman et al., evaluated a population of newly diagnosed MM patients who were eligible for chemotherapy, and showed that in a total of 187 patients, 50% reported fatigue, with 19.3% of cases being classified as severe. Among those patients treated intensively, this complaint was observed in 100% of individuals and fatigue was associated with a drop in performance.^(10)^

A recent systematic review published by Rogers et al., highlighted as important the evidence that fatigue and pain are the symptoms that most impact the quality of life of patients with myeloma.^(11)^

Given the particularities of myeloma and its treatment, such as pain complaints, difficulty in walking, recurrent infections and even incurability. Despite gains in survival with new therapies, some instruments used in oncologic routine for quality of life assessment*, i.e*, those considering patients’ symptoms and limitations, need to be individualized according to the specificities of each group of patients. Among these instruments, one of them is the Functional Assessment of Cancer Therapy - General (FACT-G).^(12)^

The FACT-G is a psychometric measurement instrument published in 1993 that was translated into several languages. This instrument aims at assessing the quality of life in cancer patients. It is a test widely used in clinical practice as well as in publications that focus on quality of life. The preference of many professionals for this test is due to its format, which has the goal to maintain the sensitivity of the test that is often quick to be completed by patients. In addition, this instrument has been subdivided over the years to be adapted to differences of each neoplasm subtype.^(12,13)^

Functional Assessment of Cancer Therapy - Multiple Myeloma (FACT-MM) is the name used for the instrument designed for MM that was developed using a structured and interactive process. The FACT-MM is composed by a literature review, according to the opinion of experts and patients, and this aims to identify the components that affected the quality of life of this neoplasia, such as, *e.g.*, limitations triggered by bone pain.^(13)^

The FACT-G has its translation and adaptation into Portuguese, as well as some of its other subscales, but, so far, its complementary approach to MM has not be translated to Portuguese language. The use of measurement scales aims to create a form of measurement that reduces subjectivity and allows assessment, interpretation and follow-up in an interdisciplinary manner. For this reason, the process of translation and adaptation of an instrument and its subscales requires the following to cross-cultural adaptation methodology.^(14,15)^

The cross-cultural adaptation process was created with the intention to maintain the instrument equivalence in another language and culture. It consists not only of a translation performed by experienced professionals, but also in retro translation of the translated version into the language of origin, followed by its review by a specialized committee, and its application as a test. Consequently, the instrument can then be assessed for equivalence and finally applied to clinical settings.^(15)^

## OBJECTIVE

To describe the process and methodology used in the translation from English into Portuguese and cross-cultural adaptation of specific subitems (MM1 and LEU3) for multiple myeloma of the Functional Assessment of Cancer Therapy - Multiple Myeloma quality of life instrument.

## METHODS

The original English version of the FACT-MM subscale was developed and validated and made available by the Functional Assessment of Chronic Illness Therapy (FACIT) Group at https://www.facit.org/. After registration and approval by this organization, the process of translation and adaptation of the subitems (MM1 and LEU3) of the FACT-MM subscale was performed according to the methodology proposed by Bonomi et al., this methodology were followed and supervised by the FACIT Group.^(16)^

The FACT-G has 27 items that are subdivided into four domains of quality of life: physical well-being (seven items), social/family well-being (seven items), emotional well-being (six items), and functional well-being (seven items). In addition, there are 14 items associated with the FACT-MM subscale, which can sometimes be shared with other subtypes of neoplasms. For each item, there is a numerical scale from zero to four, where zero means none and four means very much. The answers to the 27 items of the FACT-G will be added to the 14 items belonging to FACT-MM, in which, the higher the sum the better the patient’s quality of life.^(12,13,17)^

In the proposed MM subscale, 27+14 items are associated. Of these two parameters were not previously translated, according to the methodology: “I have trouble walking because of pain” (MM1) and “I feel discouraged about my illness” (LEU3). The LEU3 parameter is also foreseen in the subscale for leukemia patients and, at the date of conducting of this study, this had not been previously translated, unlike the other remaining 12 items.

The following items of the FACT-MM subscale “I have trouble walking because of pain” (MM1) and “I feel discouraged about my illness” (LEU3) were sent for two independent translators.

The initial translation was done by two Brazilian translators, and, at the end, there was a process of reconciliation, followed by the retrotranslation into English also conducted by two Brazilian translators. Finally, two versions were submitted to the review of four independent bilingual experts who approved and verified if the Brazilian Portuguese version was also appropriate to the European Portuguese.

After submission to the Ethics Committee of the intitution *Faculdade de Medicina do ABC* (opinion number: 554,669; CAAE: 18726314.0.0000.0082), the FACT-MM translated from English into Portuguese was randomly applied to 10 patients with a confirmed diagnosis of MM.

The selected participating patients were followed-up in a Brazilian university outpatient clinic in the ABC region, a district of the city of Sao Paulo, that belongs to the Brazilian Public Health System (SUS - *Sistema Único de Saúde*). After participants signed the informed consent, they were asked to answer a questionnaire, which was previously sent by the institution proposing the study, namely the FACIT Group. The questionnaire included directed questions about the FACT- MM subscale, and comments on the difficulty of understanding information, event at sentence level, and the degree of relevance of the item (Annex 1).

The choice of an university outpatient clinic aimed to recruit patients who reflected the sociodemographic profile of the SUS. The collection of data from patients’ medical record including details on the staging of the underlying disease and the time for diagnosis were not part of the methodology of this study.

## RESULTS

The subitems MM1 and LEU3 were, respectively, translated and culturally adapted to Portuguese as “*Tenho dificuldade em andar por causa de dor*” and “*Sinto desanimado/a em relação à minha doença*”. These sub-items were forwarded, and a consensus was conducted to the proposed methodology, their first version were accepted both by the Brazilian and Portuguese experts and then these were recognized by the FACIT Group.

After drafting, reviewing, and approving the proposed translation of FACT-MM subitems MM1 and LEU3, the cross-cultural adaptation process moved on to the testing phase, in which the forwarded questionnaire (Appendix 1) was randomly applied to 10 patients diagnosed with MM.

The population of MM patients who answered the questionnaire was characterized by equal distribution between genders, aged between 51 and 75 years (median 61 years), and eight of them were under treatment at the time of the survey. Of the total, 9 patients reported in the performance analysis provided by the questionnaire that they had some performance alteration/symptoms, and two mentioned to be bed rest.

**Figure 1:**
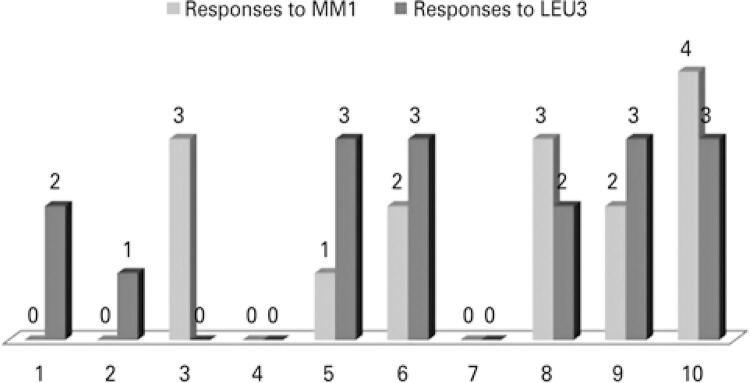
Application of the subitems “I have trouble walking because of pain” (MM1) and “I feel discouraged about my illness” (LEU3) and their scores Response to MM1: “I have trouble walking because of pain” ; Response to LEU3: “I feel discouraged about my illness” ;1: not at all; 2: more or less; 3: very; 4: very much.1- 10: Naming the study participants.

Regarding the FACT-MM and the questions directed to the validation of its translation, none of the ten patients reported difficulty in understanding the translated subitems, they did not find the content irrelevant or offensive, and they did not suggest any changes. Regarding the answers to each translated sub-item, from the total of patients, 6 reported some degree of discouragement about their disease (LEU3) and seven reported difficulty in walking because of the pain (MM1) (Figure 1).

After application, all questionnaires were electronically saved, so that they could be evaluated by the responsible institution for the instrument and, then, the translation were considered accurate and culturally adapted to the Portuguese language.

## DISCUSSION

Despite the gains in responses with new therapies developed in recent years, MM is still an incurable and extremely symptomatic disease. Reasons are either by disease-related symptoms, such as bone pain, or general symptoms such as fatigue. The patient with myeloma acquires a chronicity profile with constant losses related to his/her quality of life.^(13,16-18)^

A study carried out by Sherman et al., described 59% impairment in activities of daily life. Of these, 58% of individuals had moderate pain and 80% of patients diagnosed with MM and who were eligible for transplantation presented fatigue.^(19)^Mols et al., in a prospective populational study, assessed quality of life over a 10 years period in patients with MM. They reported impairment of quality of life throughout this period among patients with MM compared with the general population.^(20)^

The knowledge of the interference of symptoms in the quality of life of these patients is evident in the published literature.^(17- 20)^ In addition, a study conducted by the Mayo Clinic including 453 newly diagnosed patients with MM, between 2009 and 2014, showed, by means of quality of life scores, that symptoms such as pain and fatigue were predictive factors of survival. These factors also suggested that the adaptation of risk stratifications, which is traditionally based on laboratory data, when presented, need to be classified as higher risk symptoms.^(20)^

Quality of life in MM is a current concern and requires monitoring given that treatment priority may often be the control of symptoms, even in case of prolonged survival.^(6,8,15,19)^

To guarantee that the measurement of subjective symptoms, such as fatigue, to be consistently interpreted among different raters, there is a need to develop and validate scales of quality of life. This would allow to create a common language and an adequate data interpretation. Among the various instruments with this purpose and also recognized in the published literature, with emphasis on the support of the oncologic patient, there is the FACT-G, which was previously validated for Portuguese and adopted in clinical trials.^(14,15)^

Uwer et al., conducted a study to compare the equivalence of quality of life measurements using the European Organization for Research and Treatment (EORTC), Quality of Life Questionnaires (QLQ-C30, QLQ-CR38) and Functional Assessment of Cancer Therapy-Colorectal (FACT-C), which were applied to colorectal cancer patients. The authors emphasized the importance of FACT-G, since it is a global score that is easy to be applied to clinical research, while maintaining the ability to measure symptoms and satisfaction with daily life activities.^(21)^

Previously, Kelmer et al., compared the FACT-G with the EORTC and the QLQ-C30, which are other tests with broad practical-scientific applicability, and they found that the first presented reproducibility among obtained results.^(22)^

Therefore, in addition to the benefits associated with the reproducibility and easiness of applying the FACT- G, this is a self-completion test by patients. In the current scenario of patient-centered medicine, such a completion model is valued, as well as because it provides team work, and ensures individualization of treatment and measurement of heterogeneity of effects and reactions.^(23,24)^

The participation of patients is believed to increase their involvement in care and, consequently, in the decisions, allowing more engagement in issues related to their health, which may reflect positively in quality of care and satisfaction.^(24,25)^

King et al., published an investigation including various instruments of quality of life for oncologic patients in the context of evolutive indicators that measurements included the patient’s own response (Patient Reported Outcome Measures - PROMS). Their results showed that FACT-G fulfills this important criterion in the concept of patient-centered care, and it also maintains its equivalence to the EORTC-30.^(26)^

Furthermore, the FACT-G validation process, aiming to meet the profile of the Brazilian population, also had its translation and adaptation applied to a public service by using as sample a population of oncology inpatients and outpatients - which included the most varied cancer subgroups. For this reason, the test can be considered reliability to be adopted in Brazilian clinical trials.^(14,15)^

Regarding the safety of reproducibility, of note is that the process of translation and validation of a symptom measurement instrument must follow a method that aims to remove the subjectivity of understanding and maintain the objectivity proposed by the use of a scale. Consequently, the proposed cross-cultural adaptation methodology was rigorously followed and monitored by the proposing institution. Although it was not the objective of this study, it was possible to observe some degree of impairment of activities in nine out of ten subjects analyzed in this study.^(10,15,16,17,19)^

As numerous measurement instruments were created, the Scientific Advisory Committee (SAC) demanded that eight properties needed to be guaranteed, including a conceptual and measurement model with validity, reliability, responsiveness and interpretability, with administrative capacity, alternative forms and the object of this study, that is, the possibility of translation and adaptation to other cultures.

Therefore, the translation and adaptation into Portuguese of the FACT-G subitems specifically intended for MM was conducted according to international standards, so that it can be safely used to reproduce its original characteristics.^(27)^Additionally, it is fundamental that research in our field, particularly involving patients with MM, should rely on a quality of life scale specific for this disease and adapted to Portuguese speakers.

This study had limitations, such as the small number of patients included and the lack of validation of this translation of the FACT-MM into Portuguese, which will be the object of a later study. Our goal was to demonstrate the rigorousness of a translation process of subitems of a scale that applications benefits are previously known and widely used. In addition, this study was limited in terms of the interpretation of the responses obtained after the application of the questionnaire, since the complex of characteristics of its underlying disease was not evaluated, in order to allow correlation with the data that were obtained.

As demonstrated by the review of literature on the importance of the test and its participation in the current scenario of patient-centered care, we highlight the importance of this future validation respecting the rigorousness of the process. This is particularly important to allow the instrument to be tested and, consequently, to be widely adopted in clinical practice or in clinical trials, given the concern of negative implications for the quality of life of patients with MM that is even related to their survival curve.

## CONCLUSION

The subitems (MM1 and LEUE) of the Functional Assessment of Cancer Therapy - Multiple Myeloma were translated from English into Portuguese and cross-culturally adapted using the method proposed by the Functional Assessment of Chronic Illness Therapy Group. After the translation and validation process, there was no need for readjustments, a fact that enabled the quality of life instrument, which is widely known to be beneficial for the management of patients with multiple myeloma, to be used among our population after its formal validation.
